# Empathy Among Medical Students: An Exploratory Cross-Sectional Survey

**DOI:** 10.7759/cureus.60166

**Published:** 2024-05-12

**Authors:** Sukhmanjit S Brar, Revadi G, Ankur Joshi, Abhijit R Rozatkar, Ehsaas Bajaj, Abhijit P Pakhare

**Affiliations:** 1 Community & Family Medicine, All India Institute of Medical Sciences, Bhopal, Bhopal, IND; 2 Department of Psychiatry, All India Institute of Medical Sciences, Bhopal, Bhopal, IND

**Keywords:** patient outcomes, curriculum, psychology, medical education, empathy

## Abstract

Context

In the context of healthcare, effective communication and empathy are fundamental skills for physicians, as empathy correlates positively with patient satisfaction, compliance, treatment adherence, and lower rates of physician burnout, depression and anxiety. This study aimed to assess empathy levels and related factors among undergraduate medical students.

Methods

A cross-sectional study in a Central Indian medical institute examined empathy levels and factors associated with it among medical students, utilizing various scales and statistical analyses.

Results

This study found that while empathy levels were relatively high among undergraduate students, there was a decline as they progressed through medical education, particularly after the first year of clinical exposure. The study identified several factors associated with empathy levels, including perceived stress, emotional separation, and social support. Notably, individuals experiencing higher levels of stress and emotional separation tended to have higher empathy levels.

Conclusions

The study's findings suggest that medical education should incorporate interventions to enhance empathy, including addressing stress, providing social support, and exposing students to the emotional aspects of patient care.

## Introduction

Empathy, the ability to cognitively recognize an individual’s perspective and convey it back to them, is crucial for building patient trust, understanding patients’ experiences, and enhancing communication [1]. Empathy allows physicians to extract clinically relevant information: improving patient satisfaction, compliance, treatment adherence, and health outcomes, making it a pivotal skill for clinical practice [2-5]. Physicians with greater empathy demonstrate lower depression, anxiety, and distress (in the form of burnout) and higher professionalism, clinical competency, and quality of life (QOL) [6-8].

As clinicians specialize and accumulate clinical exposure, their levels of empathy decline due to heightened responsibilities, workplace stress, and fatigue from increased workloads [9]. Studies have linked a decline in physician empathy with reduced patient safety, diminished physician competence, and increased malpractice claims [8,10]. This growing concern has underscored the importance of assessing empathy levels in medical students, as those with higher levels of empathy are less prone to subsequent decline [11].

Empathy declines among students as they advance through medical education, and studies have attributed this trend to various factors. Empathy has been correlated negatively with emotional separation, desensitization, long and erratic working hours, harassment, and discrimination by superiors, focusing exclusively on disease processes [12-14] and positively with social support and positive role models [15,16]. The impact, generalizability, and causality remain unestablished due to a lack of quantitative studies on these factors and empathy levels.

The objective of this study was to assess the empathy levels among medical students using the Interpersonal Reactivity Index (IRI) scale and quantitatively correlate empathy levels with gender, preferred specialty, Perceived Stress Scale (PSS), Emotional Separation Scale (ESS), and Multidimensional Scale of Perceived Social Support (MSPSS) scores.

## Materials and methods

Study design and settings

A cross-sectional study was conducted in a medical institute in Central India.

Study participants, inclusion criteria and consent 

Medical students from the institute who consented were included in the study and administered a web-based questionnaire (see Appendices). The responses were recorded anonymously and consent for using the information for the study was taken before administration of the questionnaire.

Sampling and sampling size

Universal sampling was used for data collection, and assumptions were not made about empathy levels or their association with other factors. The institute had approximately 550 medical students, and a response rate of 40% to 50% was expected.

Study tools

The following scales were used to evaluate their respective parameters: the Interpersonal Reactivity Index (IRI) scale [17] to measure empathy, the Multidimensional Scale of Perceived Social Support [18] to measure social support, the Maintenance of Emotional Separation Scale [19] to measure emotional separation (the ability to make oneself distant, emotionally, from other individuals) and the Perceived Stress Scale [20] to measure perceived stress. Specialties were divided into two categories; clinical (medicine, surgery, pediatrics, etc.) and others (para-clinical: pathology, microbiology, pharmacology, etc.; non-clinical: anatomy, physiology, and biochemistry).

Data collection procedure

The structured self-administered questionnaire was deployed, and data was collected using an open-source web platform (eliminating interviewer bias from our study). The questionnaire comprised basic information about the participant, such as age, gender, years in medical education, preferred specialty, etc., and standardized questionnaires to measure empathy, emotional separation, social support, and perceived stress. The data was collected over two months.

Ethics

This study was conducted following the approval of the Institutional Human Ethics Committee of the institute and was designed per the Helsinki Guidelines for Informed Patient Consent of 1975 (revised in 2000).

Statistical analysis plan

Data were downloaded in Microsoft Excel (Microsoft Corporation, Redmond, WA) format, cleaned, coded, and analyzed using R-Software version 4.1.2 (R Foundation for Statistical Computing, Vienna, Austria). The continuous variables were summarized as mean with standard deviation. The correlation between empathy levels and other factors was calculated using Pearson’s correlation coefficient. The Welch ANOVA F test was used to compare mean IRI scores along the different years of medical education. The Games-Howell pairwise test was performed to compare the mean IRI scores between consecutive years. Univariate and multivariate linear regression analyses were performed to predict the IRI score with controls for confounding effects. A p-value less than 0.05 was considered statistically significant. 

## Results

Overview 

We received 211 responses (38.1% response rate). The majority of the responses were from the 1st (n=80, 37.9%) and 2nd (n=62, 29.4%) year medical students; the 3rd (n=31, 14.7%) and 4th (n=34, 16.1%) year medical students provided 30.8% responses. Table 1 shows the mean (SD) scores for the various scales included in the study. Female medical students had statistically higher empathy, perceived stress, and social support levels.

**Table 1:** Scale scores stratified by gender ^Unpaired t-test, *p < 0.05, ^1^Mean (SD), ^2^N (%) MSPSS: Multidimensional Scale of Perceived Social Support, PSS: Perceived Stress Scale, ESS: Emotional Separation Scale, IRI: Interpersonal Reactivity Index

Characteristic	Overall (n=211)	Male (n=122)	Female (n=89)	p-value^
MSPSS^1^	54.5 (15.0)	52.8 (15.1)	56.8 (14.5)	0.038*
PSS^2^	NA	NA	NA	0.024*
Low	28 (13%)	13 (11%)	15 (17%)	NA
Moderate	151 (72%)	96 (79%)	55 (62%)	NA
Severe	32 (15%)	13 (11%)	19 (21%)	NA
ESS^1^	24.5 (5.6)	25.0 (5.0)	23.8 (6.2)	0.074
IRI^1^	63.6 (11.2)	62.5 (10.3)	65.2 (12.3)	0.037*

Table 2 shows the mean (SD) scores for the four sub-scales of the IRI. Female medical students had distinctly higher scores in the empathetic concern sub-scale score. The mean IRI scores increased from the 1st to the 2nd year but declined subsequently with progression in medical education (p = 0.128). The Games-Howell pairwise test showed no discernible distinction between consecutive years of medical training.

**Table 2:** IRI sub-scale scores stratified by gender ^Unpaired t-test, *p < 0.05, ^1^Mean (SD) FS: Fantasy Scale, EC: Empathic Concern, PT: Perspective Taking, PD: Personal Distress, IRI: Interpersonal Reactivity Index

Characteristic	Overall (n=211)	Male (n=122)	Female (n=89)	p-value^
FS^1^	16.2 (4.6)	15.8 (4.8)	16.6 (4.4)	0.2
EC^1^	17.5 (4.5)	17.1 (4.3)	18.0 (4.6)	0.08
PT^1^	16.2 (4.7)	15.9 (4.5)	16.6 (5.0)	0.2
PD^1^	13.8 (3.8)	13.8 (3.9)	13.9 (3.7)	>0.9

Linear regression

We performed linear regression to identify independent factors associated with empathy levels. IRI scores were considered the outcome variable, and scores of MSPSS, PSS, ESS, course, and gender were modeled separately as independent variables. Standardized diagnostic parameters were obtained by fitting the regression model on a standardized dataset. Both 95% Confidence Intervals (Cis) and p-values were computed using a Wald t-distribution approximation: 

IRI = 𝛼 + 𝛽1 (Gender - Female) + 𝛽2 (MSPSS) + 𝛽3 (PSS) + 𝛽4 (ESS) + 𝜖

Table 3 shows the results of the univariate and multivariate regression analysis. We considered independent variables with p < 0.25 for the multivariate linear regression model.

**Table 3:** Unadjusted and adjusted univariate and multivariate linear regression analysis for predicting IRI scores *p < 0.05 MSPSS: Multidimensional Scale of Perceived Social Support, PSS: Perceived Stress Scale, ESS: Emotional Separation Scale, IRI: Interpersonal Reactivity Index

Characteristic		Univariate regression			Multivariate regression		
		Beta	95% CI	p-value	Beta	95% CI	p-value
Gender	Male	NA	NA	NA	NA	NA	NA
	Female	2.6	-0.24, 5.5	0.072	2.8	0.13, 5.5	0.040*
Other scales	MSPSS	0.07	-0.02, 0.16	0.11	0.05	-0.03, 0.14	0.2
	PSS	0.41	0.19, 0.63	<0.001*	0.30	0.08, 0.52	0.008*
	ESS	0.67	0.43, 0.91	<0.001*	0.60	0.35, 0.85	<0.001*

Table 4 shows the indices of performance for the multivariate regression model. The model explained a statistically significant and moderate proportion of variance (R2 = 0.16, F (4, 238) = 11.36, p < .001, adj. R2 = 0.15). Female gender, perceived stress, and emotional separation had positive and statistically significant impacts on empathy levels in this model.

**Table 4:** Indices of performance of multivariate linear regression model AIC: Akaike information criterion, BIC: Bayesian information criteria, R2: R-square, adj: adjusted, RMSE: root mean squared error

AIC	BIC	R^2^	R^2^ (adj)	RMSE	Sigma
1832.825	1853.784	0.160	0.146	10.253	10.361

The results for each parameter of the diagnostic model, along with the parameters for their validity, indicate that the model fits and has low levels of uncertainty (low collinearity). The linearity, homogeneity of variance, and normality of residuals all were found to lie within the reference ranges.

The equation of the final model was:

IRI = 38.3 + 2.83 (Gender - Female) + 0.05 (MSPSS) + 0.3 (PSS) + 0.6 (ESS)

Other findings

We used Pearson’s correlation coefficient to calculate the correlation between IRI and other scale scores. ESS scores were correlated positively with the overall IRI scores (r=0.322) and the various sub-scales of the IRI scale, Fantasy Scale (FS) scores (r = 0.174), Empathic Concern (EC) scores (r=0.244), and Personal Distress (PD) scores (r=0.397). PSS scores were also positively correlated with IRI scores (r=0.229) and ESS scores (r=0.229).

Table 5 shows the distribution of medical students per their preferred specialty, along with the mean (SD) IRI, MSPSS, ESS, and PSS scores. A majority of medical students preferred clinical specialties (n=183, 86.7%) over non-clinical specialties, and these students also had significantly higher empathy (IRI) scores (p = 0.003). Medical students who preferred non-clinical and para-clinical specializations had higher ESS (p = 0.021) scores.

**Table 5:** Mean (SD) IRI scores by preferred specialty for undergraduates ^Unpaired t-test, *p < 0.05, # Non-clinical and para-clinical​​​​​, ^1^Mean (SD) IRI: Interpersonal Reactivity Index, MSPSS: Multidimensional Scale of Perceived Social Support, ESS: Emotional Separation Scale, PSS: Perceived Stress Scale

Characteristic	Clinical (n=183)	Others^# ^ (n=28)	p-value^
IRI^1^	64.3 (11.4)	59.1 (9.0)	0.003*
MSPSS^1^	54.7 (15.5)	53.1 (10.6)	0.4
ESS^1^	24.3 (5.7)	26.1 (4.5)	0.021*
PSS^1^	20.5 (6.3)	18.8 (4.7)	0.10

## Discussion

Overview

In our study, medical students demonstrated high empathy levels [21]. Their empathy levels were marginally higher than those previously recorded in India but lower than those recorded in Western studies [22,23]. The empathy levels of medical students increased from the 1st to the 2nd academic year but declined from the 2nd year, that is the first year of clinical exposure \onwards. This trend was statistically insignificant in our study, but previous studies have also found a decline in empathy at the beginning of clinical exposure [12].

Why empathy declines

Previous studies have attributed the decline in empathy levels in medical students to stress (due to the higher workloads), academic burden, the time-demanding nature of the field, unfavorable workplace dynamics, and acclimatization to the medical field [12-14]. Studies have also cited the lack of concrete social support, strong role models, and humanitarian aspects of the medical curriculum as contributing factors [15,16]. We measured perceived stress, social support, and emotional separation using validated self-reported questionnaires and correlated their scores with empathy levels in our study.

Perceived stress demonstrated a strong positive correlation and emerged as an independent predictor of empathy levels in our study. Several other studies have also found that individuals who experience higher degrees of discomfort show greater familiarity and a clearer understanding of the emotional states of others [24]. This may be a possible mechanism explaining the increased empathy levels in our study.

Emotional separation demonstrated a strong positive correlation and emerged as an independent predictor of empathy levels in our study. Emotional separation may reflect the cognitive aspect of empathy, the ability to understand someone’s situation without making it one’s own. Notably, cognitive empathy is highly valued and actively pursued within clinical settings.

The observed emotional withdrawal, indicative of emotional separation, may serve as a defense mechanism against personal and emotional distress in medical students, as suggested by a previous study [24]. Furthermore, our study found strong correlations between emotional separation and both perceived stress and personal distress (a sub-scale of the IRI scale), reinforcing the link between emotional separation and the psychological well-being of medical students.

Other findings

The results of our study were consistent with previous studies and supported the generalization that women are more empathetic than men. We attributed this discrepancy to the Darwinian perspective that suggests women have evolved to possess higher levels of empathy [25]. We also found a marginally positive relationship between social support and empathy levels, indicating that engaging in social interactions necessitates the development of an emotional understanding of others [26].

Medical students who preferred clinical specialties had higher empathy levels in our study, which we attributed to these students understanding the patients better, thus being more comfortable in clinical settings. This finding was supported [27,28] and refuted by various previously published studies [29-30].

Strengths and limitations

This study attempted to better understand empathy in medical students by focusing on key factors that it could be associated with. This study identified several novel factors, such as perceived stress, social support, and emotional separation, that strongly influence and correlate with empathy levels. Since our study was limited to one medical institution, the results are internally validated, but their generalizability remains unestablished. Additionally, the factors examined in the study could only account for 14.6% of the variance in empathy levels. The research scope limited our ability to investigate the direction of causality among the factors studied, and we were unable to assess the impact of elements such as time constraints, the influence of role models, and workplace abuse. 

Recommendations

Future longitudinal studies can establish causality between specific factors, and quantitative studies can determine the impact of factors unassessed in our paper. The insights gained can provide valuable information identifying strategies to promote and sustain empathy levels in medical students. Previous randomized controlled trials have employed patient-centered interventions such as storytelling, shadowing patients, and recorded videos to raise empathy levels, knowledge retention, and subject understanding in medical students. Subsequent studies can ascertain the effectiveness of these interventions, after which their integration into the medical curriculum could elevate empathy levels and contribute to higher healthcare standards.

## Conclusions

In physician-patient relationships, empathy is crucial and has undeniable advantages for both parties. The results of our study indicate that students from our institute demonstrated high levels of empathy. The empathy levels were distinctly higher in females, in students who preferred clinical specialties, and declined with subsequent clinical exposure. The scores of the Perceived Stress Scale, Emotional Separation Scale, and Multidimensional Scale of Perceived Social Support were also reliable predictors of empathy.

## Appendices

Questionnaire

Age -

Gender -

Year -

Current / Preferred Specialty -

Read each statement, then indicate how frequently the statement was true for you by assigning the corresponding number next to the statement. Where 1 represents ‘Does not describe me very well’ whereas 5 represents ‘Describes me very well’. 

**Table 6:** Interpersonal Reactivity Index (IRI) scale

Statement	1	2	3	4	5
I daydream and fantasize, with some regularity, about things that might happen to me.	-	-	-	-	-
I often have tender, concerned feelings for people less fortunate than me.	-	-	-	-	-
I sometimes find it difficult to see things from the "other guy's" point of view.	-	-	-	-	-
Sometimes I don't feel very sorry for other people when they are having problems.	-	-	-	-	-
I really get involved with the feelings of the characters in a novel.	-	-	-	-	-
In emergency situations, I feel apprehensive and ill-at-ease.	-	-	-	-	-
I am usually objective when I watch a movie or play, and I don't often get completely caught up in it.	-	-	-	-	-
I try to look at everybody's side of a disagreement before I make a decision.	-	-	-	-	-
When I see someone being taken advantage of, I feel kind of protective towards them.	-	-	-	-	-
I sometimes feel helpless when I am in the middle of a very emotional situation.	-	-	-	-	-
I sometimes try to understand my friends better by imagining how things look from their perspective.	-	-	-	-	-
Becoming extremely involved in a good book or movie is somewhat rare for me.	-	-	-	-	-
When I see someone get hurt, I tend to remain calm.	-	-	-	-	-
Other people's misfortunes do not usually disturb me a great deal.	-	-	-	-	-
If I'm sure I'm right about something, I don't waste much time listening to other people's arguments.	-	-	-	-	-
After seeing a play or movie, I have felt as though I were one of the characters.	-	-	-	-	-
Being in a tense emotional situation scares me.	-	-	-	-	-
When I see someone being treated unfairly, I sometimes don't feel very much pity for them.	-	-	-	-	-
I am usually pretty effective in dealing with emergencies.	-	-	-	-	-
I am often quite touched by things that I see happen.	-	-	-	-	-
I believe that there are two sides to every question and try to look at them both.	-	-	-	-	-
I would describe myself as a pretty soft-hearted person.	-	-	-	-	-
When I watch a good movie, I can very easily put myself in the place of a leading character.	-	-	-	-	-
I tend to lose control during emergencies.	-	-	-	-	-
When I'm upset at someone, I usually try to "put myself in his shoes" for a while.	-	-	-	-	-
When I am reading an interesting story or novel, I imagine how I would feel if the events in the story were happening to me.	-	-	-	-	-
When I see someone who badly needs help in an emergency, I go to pieces.	-	-	-	-	-
Before criticizing somebody, I try to imagine how I would feel if I were in their place.	-	-	-	-	-

Read each statement, then indicate how frequently the statement was true for you by assigning the corresponding number next to the statement. Where 1 represents ‘Does not describe me very well’ whereas 5 represents ‘Describes me very well’. 

**Table 7:** Perceived Stress Scale

Statement	1	2	3	4	5
In the last month, how often have you been upset because of something that happened unexpectedly?	-	-	-	-	-
In the last month, how often have you felt that you were unable to control the important things in your life?	-	-	-	-	-
In the last month, how often have you felt nervous and stressed?	-	-	-	-	-
In the last month, how often have you felt confident about your ability to handle your personal problems?	-	-	-	-	-
In the last month, how often have you felt that things were going your way?	-	-	-	-	-
In the last month, how often have you found that you could not cope with all the things that you had to do?	-	-	-	-	-
In the last month, how often have you been able to control irritations in your life?	-	-	-	-	-
In the last month, how often have you felt that you were on top of things?	-	-	-	-	-
In the last month, how often have you been angered because of things that happened that were outside of your control?	-	-	-	-	-
In the last month, how often have you felt difficulties were piling up so high that you could not overcome them?	-	-	-	-	-

Read each statement, then indicate how frequently the statement was true for you by assigning the corresponding number next to the statement. Where 1 represents ‘Does not describe me very well’ whereas 6 represents ‘Describes me very well’. 

**Table 8:** Maintenance of Emotional Separation Scale

Statement	1	2	3	4	5	6
I often get so emotionally involved with my friends’ problems that I lose sight of my own feelings.	-	-	-	-	-	-
When I talk with a depressed person, I feel sad myself for quite some time after the conversation.	-	-	-	-	-	-
Sometimes I get so involved in other people’s feelings; I seem to lose sight of myself from a while.	-	-	-	-	-	-
When friends describe an emotional problem, I am in touch with their feelings without becoming too emotionally involved.	-	-	-	-	-	-
After listening to a friend tell of a scary experience, I have a difficult time studying of working.	-	-	-	-	-	-
When the worries experienced by my friends concern me, I temporarily feel these worries but don’t really get upset myself.	-	-	-	-	-	-
I usually take the problems of others home with me.	-	-	-	-	-	-

Read each statement, then indicate how frequently the statement was true for you by assigning the corresponding number next to the statement. Where 1 represents ‘Does not describe me very well’ whereas 7 represents ‘Describes me very well’. 

**Table 9:** Multidimensional Scale of Perceived Social Support

Statement	1	2	3	4	5	6	7
There is a special person who is around when I am in need.	-	-	-	-	-	-	-
There is a special person with whom I can share my joys and sorrows.	-	-	-	-	-	-	-
My family really tries to help me.	-	-	-	-	-	-	-
I get the emotional help and support I need from my family.	-	-	-	-	-	-	-
I have a special person who is a real source of comfort to me.	-	-	-	-	-	-	-
My friends really try to help me.	-	-	-	-	-	-	-
I can count on my friends when things go wrong.	-	-	-	-	-	-	-
I can talk about my problems with my family.	-	-	-	-	-	-	-
There is a special person in my life who cares about my feelings.	-	-	-	-	-	-	-
I have friends with whom I can share my joys and sorrows.	-	-	-	-	-	-	-
My family is willing to help me make decisions.	-	-	-	-	-	-	-
I can talk about my problems with my friends.	-	-	-	-	-	-	-
